# Microcanonical Entropy, Partitions of a Natural Number into Squares and the Bose–Einstein Gas in a Box

**DOI:** 10.3390/e20090645

**Published:** 2018-08-28

**Authors:** Paolo De Gregorio, Lamberto Rondoni

**Affiliations:** 1 Dipartimento di Scienze Matematiche, Politecnico di Torino, Corso Duca degli Abruzzi 24, I-10129 Torino, Italy; 2 Istituto Nazionale di Fisica Nucleare (INFN), Sezione di Torino, Via P. Giura 1, I-10125 Torino, Italy

**Keywords:** time correlation functions, time reversal, parity, quantum symmetry transformations

## Abstract

From basic principles, we review some fundamentals of entropy calculations, some of which are implicit in the literature. We mainly deal with microcanonical ensembles to effectively compare the counting of states in continuous and discrete settings. When dealing with non-interacting elements, this effectively reduces the calculation of the microcanonical entropy to counting the number of certain partitions, or compositions of a number. This is true in the literal sense, when quantization is assumed, even in the classical limit. Thus, we build on a moderately dated, ingenuous mathematical work of Haselgrove and Temperley on counting the partitions of an arbitrarily large positive integer into a fixed (but still large) number of summands, and show that it allows us to exactly calculate the low energy/temperature entropy of a one-dimensional Bose–Einstein gas in a box. Next, aided by the asymptotic analysis of the number of compositions of an integer as the sum of three squares, we estimate the entropy of the three-dimensional problem. For each selection of the total energy, there is a very sharp optimal number of particles to realize that energy. Therefore, the entropy is ‘large’ and almost independent of the particles, when the particles exceed that number. This number scales as the energy to the power of (2/3)-rds in one dimension, and (3/5)-ths in three dimensions. In the one-dimensional case, the threshold approaches zero temperature in the thermodynamic limit, but it is finite for mesoscopic systems. Below that value, we studied the intermediate stage, before the number of particles becomes a strong limiting factor for entropy optimization. We apply the results of moments of partitions of Coons and Kirsten to calculate the relative fluctuations of the ground state and excited states occupation numbers. At much lower temperatures than threshold, they vanish in all dimensions. We briefly review some of the same results in the grand canonical ensemble to show to what extents they differ.

## 1. Introduction

One tenet of equilibrium statistical mechanics is that one can derive physical predictions from calculating the number of microstates of a system compatible with some macroscipic measurements that can be performed on it [1,2,3]. This implies the reduction of the number of independent variables from the order of Avogadro’s number to a very few, even just three. For microcanonic ensembles, this improvement in the economy of computation follows from the conjecture that all the said compatible microstates, not otherwise distinguishable, are equally likely to be visited. This conjecture forms the basis of all other statistical mechanics approaches, for example, the ones employing other ensembles. However, calculations in the microcanonical ensemble are sometimes more convoluted than in other ensembles. In the so-called thermodynamic limit, i.e., one in which all the extensive variables approach infinity with equal rates, the ensembles are equivalent, the relative fluctuations of many observables approach zero and there is every reason to choose the ensemble that is more practical for computation. As it has been pointed out [4,5,6,7,8], for mesoscopic quantum systems, the situation is more articulated. Experiments of Bose–Einstein condensates employ relatively few atoms [9,10,11], yet their number is sufficiently large to allow asymptotic expansion of the relevant mathematical functions. With those numbers, the fluctuations of the calculated thermodynamic variables change among different ensembles [12,13].

The grand canonical ensemble is most commonly used to treat Bose–Einstein condensates of non-interacting particles because it greatly simplifies the mathematical burden of the calculations. However, the thermodynamic (or ‘bulk’) limit is assumed beforehand [14]. This is a drawback for mesoscopic systems and the choice of which ensemble is best to use is a problematic one. Temperley [15] was among the firsts to recognize that the equivalence of ensembles in dealing with quantum systems is not always obvious. In his analysis, he contrasted the microcanonical and grand canonical ensemble approach and he pointed out that some of the implicit mathematical assumptions in the grand canonical ensemble are better suited in the low and high energy/temperature regimes, but not necessarily in the intermediate regions. However, one should bear in mind a discussion over the orthodicity of the microcanonical ensemble [1], which emerges when a continuous phase space is additionally assumed. In this respect, treating mesoscopic systems via the microcanonical ensemble implicates additional assumptions when the thermodynamic relations are obtained through derivation.

To put things into context, let us define the entropy:

(1) $$S=k_{B}\log\Omega (\underline{x}|\underline{X}),$$

where log stands for the natural logarithm, the vector $\underline{x}$ is the set of independent microscopic variables, the vector $\underline{X}$ the set of macroscopic independent variables (for example $\underline{X}=\left(V,N,E\right)$). $\Omega (\underline{x}|\underline{X})$ is the number of all states $\underline{x}$ conditional to an assignment of $\underline{X}$, and $k_{B}$ is Boltzmann’s constant. Here, we are not interested in the statistics and entropy of the interacting systems where the advanced methods of quantum-field theory, Green’s functions and Dyson’s equation (and so on) should be employed.

Clearly, $\Omega (\underline{x}|\underline{X})$ must be a positive integer, which implies a cancellation of all the dimensional quantities. This in turn implies that $\Omega (\underline{x}|\underline{X})$ is a sum of certain integers, but that sum might not be mathematically substituted by an integral always for free [15]. One (historically older) approach has its roots in classical mechanics and it prescribes that one must divide the total phase space hypervolume into small *elementary* hypervolumes. They become the units to be counted, conditioned to the $\underline{X}$ values. It must be noted that, when $\underline{X}=\left(E,V,N\right)$, i.e., energy, volume and particles’ number, there is still an ongoing debate concerning whether the microstates to be counted should be those compatible with the total energy *E* falling between a vanishingly thin hypershell around the spherical *E*-equisurface, or those of any energy up to *E* [16,17,18,19,20,21]. In this work, we shall not assess this issue; however, we have chosen to use the surface approach.

The quantum analogues of the classical calculations employ well-structured counting principles, which in the case of non-interacting particles involve the single particles’ energy spectra, later on identified by us by the symbols Λ and $\Lambda^{+}$. As in this work we shall recall, they are perfectly suited to account for the classical (ideal gas) results. The energy is given in integer units of some prescribed $\epsilon_{0}$. Each disposition is counted once, per the equal priors assumption. For example, all integers can be allotted for photons or phonons, but only squares of integers can be allotted for particles. Call $n_{k}$ such allowed integers. Then, a constraint on their sum $\sum_{k}n_{k}=m$ must be satisfied, with the total energy given by $E=m\epsilon_{0}$.

In the present work, we try to broaden the impact of the works [4,5,6,7,8], who considered microcanonic entropy calculations of ideal Bose gases in harmonic traps, by considering the complementary problem of ideal gases of free particles caged in *d*-dimensional regions of space (with some more details given in one dimension, however, we shall exclude two dimensions because of the many similarities with the one-dimensional harmonic traps). Furthermore, building on previous work [22], we investigate the relative fluctuations of the ground state and excited states occupancy numbers, in the microcanonical ensemble Bose–Einstein condensation theory, both in one and three dimensions. Given the rarity of results in this area which are uniform both in the energy and in the particles’ number, our aim is also to point to interesting open mathematical problems, with potential physical impact. Some of these problems are tremendously complex from the mathematical point of view. To guide the reader in this articulated scenario, we also show a few examples in which the mathematical results are satisfactorily rich, so that some known traditional physical results are easily recovered, albeit in a scholarly less expendable fashion.

To start with a more edible problem, consider a physical model in which the *N* elements are treated as distinguishable. Each of them can take up from 0 to *m* integer units of energy $\epsilon_{0}$, but the sum over the *N* elements’ ‘occupation numbers’ must equal *m*. $\Omega_{\epsilon_{0}}\left(\underline{n}|N,m\right)$ is therefore given by the number of ways that *m* items can be allotted into *N* distinct urns. This is one of the few cases in which an explicit and compact formula is available for any *N* and *m*, namely

(2) Ωϵ0(n_|N,m)=N+m−1m.

Of course, $E=m\epsilon_{0}$ holds. One, however, has to be careful as to what the zero-reference value for the energy is. Sometimes, we shall refer to $E^{*}$ when solely the system’s excitation energy is considered.

Other physical situations involve more elaborate partition sums, even for non-integracting elements. At least two key modifications can be considered. One involves the case of indistinguishable elements. As an illustrative example, set m=9 and N=3. Then,

(3) 1+2+6=9,2+6+1=9and6+1+2=9

all contribute to determining Equation (2), while they contribute only one configuration when the elements are indistinguishable. Concurrently, the sum 3+3+3=9 contributes only once both in Equation (2) and in the case of indistinguishable elements. This is essentially the problem first posed by Bose in physical terms [23], and the problem of partitions vs. compositions in mathematical terms. It is also the partition problem considered in the works dealing with one-dimensional harmonic traps [4,5,6,24].

The other modification concerns the set of allowed integers in each sum. Equation (2) is a consequence of having chosen non-negative integers as summands. When the elements are independent particles, each with given momenta, then the only allowed values are squares of non-negative integers. For example, $1+1+4=1^{2}+1^{2}+2^{2}=6$ becomes the only allowed partition into squares of m=6 with N=3, for indistinguishable particles, while in turn it counts the only three allowed compositions for distinguishable particles (by permuting the order).

Despite the simplicity of the formulations of the problems of such kind, not all of them have been completely solved. We shall try to spell out some of their hidden subtleties. In some cases, we shall consider the effect of substituting the partition sums by integrals, which at some stage is the most common simplification adopted in calculations in the Physics community. This is already widely covered even in textbooks on the subject, thus we only touch upon it. There are, however, plenty of results in the mathematical community and in number theory dealing with asymptotic (mainly large *m*) estimations of the number of partitions or compositions of an integer into a sequence of other assigned integers [25,26,27,28,29,30,31,32,33], many of which have direct physical applications [4,5,6,7,8,34,35,36]. In the case of partitions of *m* into integers, it was only in relatively recent times that decades-old mathematical result have been exploited to calculate thermodynamic relations, namely for Bose–Einstein particles trapped in a one-dimensional harmonic potential [4,5,24].

We have discovered an interesting result in number theory, dating back to more than 60 years ago [37], which to our knowledge has not been discussed in the Physics community. It deals with the number of partitions of *m* into the sum of *N* squares of positive integers, when *m* is arbitrarily large and *N* grows with *m* in some ways. We argue that it can be used to derive the entropy of a Bose–Einstein gas of independent particles, caged in a one-dimensional line with hard walls. Therefore, to derive the relation between energy and temperature, far deep into the quantum regime and, to some extent, around the critical region. We use results in [37] also to find the entropy of a Bose–Einstein gas contained in a three-dimensional box, confirming the correct asymptotic result. We then apply the results in [22] to characterize the fluctuations of the excited states in all dimensions, finding how they depend on the dimensionality. We stress that other interesting physical predictions might be deduced using variations of the set of allowed integers in the partition sums. We do not suggest that the physical regimes that would discriminate between our approach and others are comfortably attainable experimentally. Nevertheless, some other, more foundational theoretical questions may be attacked, exploiting some of our observations. We also suggest that there is a unique formal expression for the entropy of a non-interacting gas, as derived from the microcanonical ensemble, which spans the entire range of temperatures, from close to the ground state to the ideal classical gas. The result depends on a intuitive mathematical conjecture that we propose.

In this article, we shall refer to O(x) for any quantity such that $\lim_{x\to \infty }\frac{O(x)}{x}=const\neq 0$. We shall refer to o(x) for any quantity such that $\lim_{x\to \infty }\frac{o(x)}{x}=0$. The symbol ∼ will stand for equalities which are true at least to *o*-order of the quantity on the right-hand side, in the declared reference (asymptotic) variable. The symbol ∝ will stand for ‘proportional to’.

## 2. Partitions and Compositions: The Case of Integers

We first need to formalize mathematically the (non-interacting) single-particle energy spectra. Therefore, we denote with $\Lambda^{+}$ any sequence of positive integers, $1\leq \lambda_{1}\leq \lambda_{2}\leq \lambda_{3}\leq \ldots$, and with Λ the associated sequence of non-negative integers, $0=\lambda_{0}\leq \lambda_{1}\leq \lambda_{2}\leq \ldots$. We call unrestricted partition of *m* on Λ (or on $\Lambda^{+}$) any sum such that

(4) $$\lambda_{k_{1}}+\lambda_{k_{2}}+\lambda_{k_{3}}+\cdots +\lambda_{k_{r}}=m,\;\mathrm{with}\;\lambda_{k_{1}}\leq \lambda_{k_{2}}\leq \lambda_{k_{3}}\leq \cdots \leq \lambda_{k_{r}}\in \Lambda \;\;\;\left(\mathrm{or}\;\in \Lambda^{+}\right).$$

Given *m*, the total number of its unrestricted partitions is denoted by $p_{\Lambda }\left(m\right)$ (correspondingly $p_{\Lambda^{+}}\left(m\right)$).

Since unrestricted partitions can be viewed to contain an arbitrary number of zeros, it follows that $p_{\Lambda }\left(m\right)=p_{\Lambda^{+}}\left(m\right)$. If additionally $\lambda_{k}=k$, the index is usually dropped and $p_{\Lambda }\left(m\right)=p\left(m\right)$ denotes the number of partition of *m* into positive integers. Hardy and Ramanujan were the first to attack this problem thoroughly [38]. Here, we report their well known asymptotic result:

(5) $$p\left(m\right)∼\frac{e^{\;C\sqrt{m}}}{4m\sqrt{3}}\;,\;C=\pi \sqrt{\frac{2}{3}}.$$

This quantity was considered by various authors [4,5,6,15] as a starting point to deal with bosonic quantum systems, specifically it is relevant for bosons in one-dimensional harmonic traps.

We mention that the expected number of summands in p(m) is asymptotically given by [22]

(6) $$\bar{r}\left(m\right)=C^{-1}\sqrt{m}\log m+2C^{-1}\left(\;\gamma +\log\left(2C^{-1}\right)\;\right)\sqrt{m}+O\left(\log m\right)\;.$$

We define restricted partition of *m* a partition like the one in Equation (4), in which *r* (the number of summands) is assigned. We denote it as $p_{\Lambda }\left(r,m\right)$ or $p_{\Lambda^{+}}\left(r,m\right)$, depending on the assigned sequence. Now $p_{\Lambda }\left(r,m\right)$ and $p_{\Lambda^{+}}\left(r,m\right)$ differ, but $p_{\Lambda }\left(r,m\right)$ has the alternative interpretation of denoting the number of partitions of *m* with *up to r* strictly positive summands. Additionally, when $\lambda_{k}=k$, then $p_{\Lambda }\left(r,m\right)=p_{\Lambda^{+}}\left(r,m+r\right)≔p^{*}\left(r,m\right)$, which denotes the number of partitions of *m* with up to *r* positive integers as summands. The stated equality can be understood with some elementary reasoning. Still, when $\lambda_{k}=k$, we further denote $p\left(r,m\right)≔p_{\Lambda^{+}}\left(r,m\right)=p_{\Lambda }\left(r,m-r\right)$, the number of partitions of *m* with exactly *r* positive integers as summands. We present some asymptotic known results, the first of which is due separately to Erdös and Lehner [39] and to Auluck, Chowla and Gupta [40], albeit in slightly different forms, which we present here as

(7) $$p^{*}\left(r,m\right)∼p\left(m\right),\;\mathrm{if}\;r=C^{-1}\sqrt{m}\log m+x\sqrt{m}\;,$$

(8) $$\frac{p^{*}\left(r,m\right)}{p(m)}∼0,\;\;\;\;\mathrm{if}\;r=C^{-1}\sqrt{m}\log m-x\sqrt{m}\;,$$

where $x\in R^{+},\;x\gg 1$ and ∼ refers to asymptotically in *m*. When *x* is any positive real, not necessarily large *m*-independent number, Erdös and Lehner [39] report a more precise result as a function of every *x*, which has been exploited by others [4,5,6,15,24] to predict the behavior of some Bosonic systems at low temperature. The above result (7) and (8) predates that, in Equation (6); however, it can be interpreted by comparison with Equation (6), if we suppose that, asymptotically in *m*, the distribution of *r* values in the partitions counted by p(m) is sharply peaked around its maximum. Therefore, $\bar{r}\left(m\right)$ defined in Equation (6) is proximate to the optimal number of summands. Thus, p(r,m) is much smaller than p(m) if *r* is much less than optimal, while it is almost equal to p(m) if *r* is much larger than optimal. The latter case may be realized, for example, when r=O(m). Finally, since $p\left(r,m\right)=p^{*}\left(r,m-r\right)$, then when $r=O(m^{\frac{1}{2}+\epsilon })$, ϵ>0,

(9) $$p\left(r,m\right)∼p\left(m-r\right)∼\frac{e^{\;C\sqrt{m-r}}}{4\left(m-r\right)\sqrt{3}}\;,$$

which, if *r* is not of order *m*, implies the leading correction in *r* to p(m) to be $∼\exp\left(-\frac{Cr}{2m}\right)$. As we shall see, the existence of a region in which the number of summands is optimal, for the partition of *m* of physical interest, is what characterizes the critical region of many Bose–Einstein systems, whether or not a genuine phase transition exists. It is the threshold for the energy below which a macroscopic number of particles occupies the ground state. Essentially, the first moments calculated in [22] for several partitions problems offer a straightforward recipe to identify those thresholds. In the case of p(r,m), from [22], one can derive that the second central moment of *r* increases logarithmically with *m*, a measure of the dispersion of *r* around the value $\bar{r}\left(m\right)$ in Equation (6) for the unrestricted partitions.

Not much is known beyond Equation (8) about p(r,m) and $p^{*}\left(r,m\right)$, when *r* lies in the range between being $O(m^{\frac{1}{3}})$ and $O(m^{\frac{1}{2}})$. However, the following holds when $r=o(m^{\frac{1}{3}})$ [40]:

(10) p(r,m)∼1r!m−1r−1.

Below, we shall see that it is easy to convince oneself that the right-hand side of Equation (10) is a lower bound for the left-hand side, for any *r*. The nontrivial result lies in the associated asymptotics. Furthermore, to the leading order, the same expression was proved to hold with p(r,m) replaced by $p^{*}\left(r,m\right)$, i.e., [39]

(11) p*(r,m)∼1r!m−1r−1.

In fact, by using the dominant term in Stirling’s approximation, i.e., logk!∼klogk, and noticing that Equation (10) also implies

(12) p*(r,m)∼1r!m+r−1r−1,

then Equations (10) and (11) are both consistent with

(13) $$\log p(r,m)∼\log p^{*}\left(r,m\right)∼r\log\frac{m}{r^{2}}\;,$$

which we can also interpret to imply that, if $r=o(m^{\frac{1}{3}})$, then $p^{*}\left(r,m\right)$ is dominated (asymptotically in *m*) by the contribution of the partitions with exactly *r* summands, while partitions with less summands can be neglected.

We conclude this first part by noticing that $p_{\Lambda^{+}}\left(r,m\right)$ also represents the number of solutions of the following Diophantine equation:

(14) $$\begin{array}{c} \left\{\begin{array}{c} x_{1}+x_{2}+\ldots \;\;=r,\;\;\;\\ x_{1}\lambda_{1}+x_{2}\lambda_{2}+\ldots =m\;, \end{array}\right. \end{array}$$

where $x_{i}$’s are all non-negative integers. The sums have nominally infinite terms, but only finite and upper-bounded positive terms.

When the order of summands is relevant, we define the homologous counting functions for compositions, namely $\rho_{\Lambda }\left(m\right)$, $\rho_{\Lambda^{+}}\left(m\right)$, $\rho_{\Lambda }\left(r,m\right)$ and $\rho_{\Lambda^{+}}\left(r,m\right)$. In this work, we only consider the case of restricted compositions. For $\lambda_{k}=k$, we naturally define the number of compositions of *m* into, respectively, exactly *r* or at most *r* summands, by ρ(r,m) and $\rho^{*}\left(r,m\right)$. Thus,

(15) ρ(r,m)=m−1r−1.

Concurrently,

(16) ρ*(r,m)=m+r−1m=m+r−1r−1,

which is now an interpretation of Equation (2), with r=N.

To see that r!p(r,m)≥ρ(r,m), which we anticipated after stating Equation (10), simply consider the best case scenario of a partition whose summands are all different. For that occurrence, the same partition originates r! distinct compositions, so the equality stands. In all other partitions, r!p(r,m) is an overestimation of the compositions adding up to *m*. The inequality tends to be strong when *r* is very large, while it tends to be weak when *r* is suitably smaller than *m*. Similar considerations apply to Equations (15) and (16), in the sense that they lead to essentially coincident asymptotic descriptions only for moderately large values of *r*; otherwise, they differ considerably.

## 3. Partitions and Compositions with Integers: The Physics and a Comparison with Integrals

Arguably, the first physicist to conceive of the use of the Formulas (2)–(16) was Max Planck, in his very first modern derivation of the emission spectrum of a black body [41], and it is still used today to derive physical laws [42]. Incidentally, it did not involve a microcanonical ensemble, since Planck was dealing with classical radiation of light. Rather, he considered the number of ways a collection of emitters could radiate at a given frequency, to which he then assigned a Boltzmann distribution. More generally, photons are not suitable for treatments in which the number of elements is fixed, since their number fluctuates in a real context, even for closed systems. Nevertheless, as Planck demonstrated, counting the number of realizations with both *N* and energy fixed can be incorporated into the calculation at some stage. Phonons in one dimension can be treated in an equal manner. However, the utility of the present analysis lies more in the simplicity of the calculations involved, which help clarify some basic issues associated with the use of either discrete or continuous analysis. It is in fact for this very reason that quantum mechanics entered into the scientific scene for the first time.

We shall consider a closed system with *N* elements. Each element can have any number of energy units $\epsilon_{0}$, from 0 to *m*, and the only restriction is that the sum of energy units over all the elements must be equal to *m*. Then, Equation (2) can be used, which is also Equation (16) with r=N. $m=\frac{E}{\epsilon_{0}}$, where *E* can be taken as the excitation energy, by simply defining its zero reference value to be in a one-to-one correspondence with that in which all the *N* particles have zero units of $\epsilon_{0}$. Both *N* and *m* are very large, therefore Stirling’s approximation can be used and the entropy $S/k_{B}=\log\;\rho^{*}\left(N,m\right)$ is

(17) $$S/k_{B}=\left(m+N\right)\log\left(m+N\right)-m\log m-N\log N\;.$$

Defining m=yN and S=Ns,

(18) $$s/k_{B}=\left(1+y\right)\log\left(1+y\right)-y\log y\;,\;\;y=\frac{E}{N\epsilon_{0}}\;.$$

This is a well defined entropy, which can be approximated by

(19) $$s/k_{B}=1+\log y\;\mathrm{if}\;y\gg 1$$

and by

(20) $$s/k_{B}=y-y\log y\;\mathrm{if}\;1\gg y\;.$$

Calculating the entropy from an integral is permitted only via the definition of some dimensional energy scale $\epsilon_{0}^{\prime }$. First, one has to calculate the normalized hypervolume V comprising all states with energy less than *E*, i.e.,

(21) $$V=\frac{\int_{0}^{E}d\epsilon_{1}\int_{0}^{E-\epsilon_{1}}d\epsilon_{2}\cdots \int_{0}^{E-\epsilon_{1}-\ldots -\epsilon_{N-1}}d\epsilon_{N}}{\epsilon_{0}^{\prime \;N}}=\frac{1}{N!}{\left(\frac{E}{\epsilon_{0}^{\prime }}\right)}^{N}\;.$$

Then, Ω is defined as the hypeshell of small thickness $\delta_{\epsilon }$ by first derivation of V:

(22) $$\Omega =\frac{\partial V}{\partial E}\delta_{\epsilon }=\frac{1}{(N-1)!}{\left(\frac{E}{\epsilon_{0}^{\prime }}\right)}^{N-1}\frac{\delta_{\epsilon }}{\epsilon_{0}^{\prime }}\;.$$

At this point, assumptions must be made. If the dimensional scale $\epsilon_{0}^{\prime }$ and the infinitesimal thickness $\delta_{\epsilon }$ should depend on *N* in some unknown manner, then the calculation is a futile exercise. It is intuitive that $E\gg \epsilon_{0}^{\prime }$ must hold. If $\epsilon_{0}^{\prime }$ is also independent of *N*, then $\delta_{\epsilon }=\epsilon_{0}^{\prime }$ can be assumed without too much loss of generality. By dropping the subtraction of 1, we can write

(23) $$\Omega ≃\frac{1}{N!}{\left(\frac{E}{\epsilon_{0}^{\prime }}\right)}^{N}=V\;.$$

If $N!\;\epsilon_{0}^{\prime \;N}>E^{N},$ we arrive at the unphysical conclusion that there is less than one state available. From Stirling’s approximation, this is equivalent to $N\epsilon_{0}^{\prime }>E$. For the method to hold in general, the quantity $\epsilon_{0}^{\prime }$ should be assumed ever so small that $E\gg N\epsilon_{0}^{\prime }$ always holds, whatever the number *N*. We arrive at

(24) $$s/k_{B}=\log y^{\prime }=\log y+\log\frac{\epsilon_{0}^{\prime }}{\epsilon_{0}}\;,\;\;y^{\prime }=\frac{E}{N\epsilon_{0}^{\prime }}\;\left(y^{\prime }\gg 1\right)\;,$$

which is consistent with the approximation (19). The limitation of the continuous approach is obvious, since the trend represented in Equation (20) is inaccessible by this method of calculation. If the elements are harmonic oscillators, $\epsilon_{0}=\hbar \omega$ sets a lower bound for $\epsilon_{0}^{\prime }$.

So far, we have treated the *N* elements as distinguishable. If they are not, we shall assume a boson-like behavior. Then, N=r and we can take advantage of either Equation (7) or Equation (11), as appropriate according to the context. *m* designates the units of excitation energy, $E^{*}=m\epsilon_{0}$ and $S/k_{B}=\log\;p^{*}\left(N,m\right)$. By virtue of the asymptotic equation (7), $N\gg \sqrt{m}$ implies

(25) $$S/k_{B}=C{\left(\frac{E^{*}}{\epsilon_{0}}\right)}^{\frac{1}{2}}-\log\frac{E^{*}}{\epsilon_{0}}\;,$$

which is independent of *N*. By adding the estimated (weak) dependence on *N*, this approach can be improved to calculate the entropy of a closed system of exactly *N* bosons trapped in a one-dimensional harmonic potential, with $\epsilon_{0}=\hbar \omega$ [4]. The critical interest in this approach stemmed from the study of mesoscopic systems, since the often used alternative approach of employing the grand canonical ensemble suffers from the standard underlying assumption that the energy, the size and the number of particles approach infinity, with equal rates [14]. In fact, the finite-size effects can be thoroughly investigated in the grand canonical ensemble [7,8]. In the microcanonical ensemble, the number of particles is the independent variable and through number theory it is possible to compute the finite size (or finite-*N*) effects and to compare them with other ensembles [6].

We mention that, from Equation (6), with $\bar{r}\left(m\right)$ reinterpreted as $N_{c}\left(E\right)$ (the critical number of particles which dominates the partition measure), together with Equations (7) and (8) and the physical entropic interpretation (25), it is possible to define a critical threshold temperature, $T_{c}$, below which a macroscopic number of particles is expected to occupy the ground state. We mention this here because we shall often recur to such arguments in the next sections. To simplify things, we shall adopt standard first derivations rather than finite-difference equations.

We shall momentarily drop the dimensional constants. From $N_{c}\propto E^{\frac{1}{2}}\log E$, and since $T^{-1}=k_{B}\frac{dS}{dE}=k_{B}\frac{dS}{dE^{*}}$, then when $N\gg N_{c}$ Equation (25) informs us that $E^{*}\propto T^{2}$. It is straightforward to obtain $N_{c}\propto T\log T$. The leading factor for the critical (crossover) temperature is therefore $T_{c}\propto \frac{N}{\log N}$.

The next computable region with exact results available is when $m\gg N^{3}$, in which case the asymptotic identity (11) holds. Use of the approximation (13) is legitimate here and, with *y* defined in alongside Equation (18), it implies:

(26) $$s/k_{B}=\log y-\log N\;.$$

Clearly, we are dealing with systems not restrained by the requirement of the entropy being extensive. Nevertheless, since $y=\frac{m}{N}\gg N^{2}$, the −logN term might be unimportant. We also have to be reminded that $m\gg N^{3}$ is a condition between integer numbers, and one is not forced to rule out that as long as it is satisfied, the entropy as a function of *y* is a legitimate quantity.

As for the case of distinguishable elements, in this ‘high energy’ region, we arrive at the same conclusions via simple integration. We substitute the integral (21) with one that has the additional constraint that the energies are ordered in an increasing fashion. To do so, first we define

(27) $$H\left(x_{1},x_{2},\ldots ,x_{N}\right)=\left\{\begin{array}{c} 1,\;\mathrm{if}\;x_{1}\leq x_{2}\leq \ldots \leq x_{N},\\ 0,\;\mathrm{otherwise}, \end{array}\right.$$

and then

(28) $$V=\frac{\int_{0}^{E}d\epsilon_{1}\int_{0}^{E-\epsilon_{1}}d\epsilon_{2}\cdots \int_{0}^{E-\epsilon_{1}-\ldots -\epsilon_{N-1}}d\epsilon_{N}\;H\left(\epsilon_{1},\epsilon_{2},\ldots ,\epsilon_{N}\right)}{\epsilon_{0}^{\prime \;N}}=\frac{1}{{\left(N!\right)}^{2}}{\left(\frac{E}{\epsilon_{0}^{\prime }}\right)}^{N}\;.$$

From here, one can easily verify that $\epsilon_{o}^{\prime }=\epsilon_{0}$ implies Equation (26). We see the emergence of an additional Gibbs-like N! in the denominator. Contrary to conventional situations, this factor does not ‘fix’ the extensivity of entropy, rather it impedes it in the ‘classical’ regime. One may argue that, since the energy is very large, then at least the length-scale of the container must be considered at some point, and the classical limit remains abstract. We shall see that this situation is different when massive non-interacting particles or bosons are considered, leaving some room open to the prospect of having a unique entropy expression, spanning the whole range of energy, and consistent with both classical and quantum physics.

## 4. Partitions and Compositions: The Case of Squares of Integers

More than 60 years ago, Haselgorve and Temperley studied [37], among others, the problem of calculating the number of partitions of an integer *m* into the sum of *r* positive squares, solving the problem asymptotic in *m* (with *r* growing in some way with *m*). This is equivalent to solving the system (14), with $\lambda_{k}=k^{2}$. We shall use the subscript *s*, to symbolize ‘squares’, to indicate the partitions and compositions of interest. Thus, $p_{s}\left(r,m\right)$ is the number of restricted partitions of *m* with exactly *r* positive integer squares as summands, $p_{s}\left(m\right)$ the number of unrestricted partitions of *m* into squares, $p_{s}^{*}\left(r,m\right)$ the number of partitions with up to *r* positive integer squares as summands (or equivalently, with exactly *r* non-negative integer squares). The solution of the corresponding unrestricted problem was also proposed by Hardy and Ramanujan [38], taken up by Wright [43] and thoroughly investigated by Vaughan [32]. Haselgrove and Temperley [37] considered sets $\Lambda^{+}$ that satisfied specific conditions, among which that the sum $\sum_{k}\lambda_{k}^{-2}$ be convergent. They used the generating function

(29) $$g\left(x,z\right)=\prod_{k=1}^{\infty }{\left(1-xz^{\lambda_{k}}\right)}^{-1}=\sum_{r=0}^{\infty }\sum_{m=0}^{\infty }p_{\Lambda^{+}}\left(r,m\right)x^{r}z^{m}\;,$$

and for $\lambda_{k}=k^{2}$ obtained:

(30) $$\log p_{s}\left(m\right)∼\;3Am^{\frac{1}{3}}\;,$$

(31) $$p_{s}\left(r,m\right)∼Am^{-\frac{2}{3}}f\left(r\;Am^{-\frac{2}{3}}\right)p_{s}\left(m\right),$$

(32) $$f\left(x\right)=\left\{\begin{array}{cc} 2\sum_{k=1}^{\infty }{\left(-1\right)}^{k+1}\;k^{2}\;e^{-x\;k^{2}}, & \mathrm{if}\;\;x>0\;,\\ 0, & \mathrm{if}\;\;x\leq 0\;, \end{array}\right.$$

(33) $$A={\left[\frac{1}{2}\;\Gamma \left(\frac{3}{2}\right)\;\zeta \left(\frac{3}{2}\right)\right]}^{\frac{2}{3}}=1.102\ldots \;.$$

Γ is Euler’s gamma and ζ in Riemann zeta. The function f(x), seen as a distribution, has a maximum smaller than its mean. When *r* is of the order of $m^{\frac{2}{3}}$, the quantity $m^{\frac{2}{3}}p_{s}\left(r,m\right)$ is of the order of $p_{s}\left(m\right)$. When *f*’s argument *x* is moderately larger than 1, $p_{s}\left(r,m\right)$ becomes (in *r*) exponentially smaller than $p_{s}\left(m\right)$. If x≫1, we can approximate $f(r\;Am^{-\frac{2}{3}})$ with $2\;e^{-r\;Am^{-\frac{2}{3}}}$, and we obtain:

(34) $$\log p_{s}\left(r,m\right)∼\;3Am^{\frac{1}{3}}-r\;Am^{-\frac{2}{3}}+\log m^{-\frac{2}{3}}+\log\left(2A\right)\;.$$

We must caution the reader that this result is not consistent with the requirement that $p_{s}\left(m,m\right)=1$. It suffices to substitute r=m in Equation (34) to verify it. In fact, the calculation leading to Equation (31) is asymptotic in *m*, namely it neglects contributions of the type $o\left(m^{-\frac{2}{3}}p_{s}\left(m\right)\right)$, which may become important when *r* approaches values comparable with *m*. Equation (31) can be matched with iterated difference equations in *r* and *m*. It will be useful for our purposes to recall the following ones:

(35) $$\begin{array}{c} p_{s}\left(r,m\right)-p_{s}\left(r,m-1\right)∼A^{2}m^{-\frac{4}{3}}f\left(r\;Am^{-\frac{2}{3}}\right)p_{s}\left(m\right)\;, \end{array}$$

(36) $$\begin{array}{c} p_{s}\left(r,m\right)-p_{s}\left(r-1,m\right)∼A^{2}m^{-\frac{4}{3}}f^{\prime }\left(r\;Am^{-\frac{2}{3}}\right)p_{s}\left(m\right)\;, \end{array}$$

which are again valid provided, respectively, that *f* and $f^{\prime }$ are not o(1) in *m*, where $f^{\prime }$ is the first derivative of *f* with respect to its argument.

Another striking result of Haselgorve and Temperlay [37] is that they derived partial results in more generality, for any sequence of the type $\lambda_{k}=k^{\;\delta }$, δ>1, then, if q=δ+1,

(37) $$p_{\Lambda^{+}}\left(m\right)∼q\;A_{\delta }\;m^{\frac{1}{q}},\;\;A_{\delta }={\left[\frac{1}{\delta }\;\Gamma \left(\frac{q}{\delta }\right)\;\zeta \left(\frac{q}{\delta }\right)\right]}^{\frac{\delta }{q}},$$

which is thoroughly examined also in [33]. Haselgrove and Temperley also offer hints on how to estimate $p_{\Lambda^{+}}\left(r,m\right)$ for such sequences. Their analysis is less articulated than for the case δ=2 just presented above. Conversely, it is slightly more general because, in fact, they considered sequences such that $N∼\lambda^{\frac{1}{\delta }}$, where N(λ) is the number of terms in the succession $\Lambda^{+}$ that are ≤λ. The distribution *f* in the asymptotic identity (31) is substituted by a function with similar properties, which has a maximum around $r=O(m^{\frac{\delta }{q}})$.

For reasons that will be clear in the next section, here we are interested in considering the following sequence:

(38) $$\lambda_{k}=k^{2}+2k,\;i.e.,\;\;\Lambda^{+}=\left\{3,\;8,\;15,\;24,\ldots \right\}\;$$

for which we shall use the conventions that $p_{\sigma }\left(\cdot \right)=p_{\Lambda^{+}}\left(\cdot \right)$ and $p_{\sigma }^{*}\left(\cdot \right)=p_{\Lambda^{+}}^{*}\left(\cdot \right)$. Notice that the sequence in Definition (38) can be obtained from the usual sequence of squares of positive integers, by simply shifting its elements one place to the left, and then subtracting 1 from each element. This leads to

(39) $$p_{s}\left(r,m\right)=p_{\sigma }^{*}\left(r,m-r\right)\;.$$

Since asymptotically the sequence (38) satisfies $N\left(\lambda \right)∼\lambda^{\frac{1}{2}}$, following [37], we conjecture that, similarly to Equation (31), and with $p_{s}\left(m\right)$ given in Equation (30),

(40) $$p_{\sigma }\left(r,m\right)∼Am^{-\frac{2}{3}}g\left(r\;Am^{-\frac{2}{3}}\right)p_{s}\left(m\right),$$

where the function g(x) is a distribution which differs from f(x), but shares its main properties, i.e., it has a maximum different from its average, both of which are in the vicinity of unity. Now, suppose that $r\gg m^{\frac{2}{3}+\epsilon }$ for positive ϵ, then by integrating the function g(x) in its range, we coherently conjecture that

(41) $$p_{\sigma }^{*}\left(r,m\right)∼p_{s}\left(m\right).$$

Thus, while, for integers, $p\left(r,m\right)=p^{*}\left(r,m-r\right)$ holds for any *r* and *m*, here, in view of Equation (39) and the conjecture (41), we are positing that asymptotically in *m* (and *r* appropriately large),

(42) $$p_{s}\left(r,m\right)∼p_{s}\left(m-r\right)\;.$$

As we shall mention in the next section, the above result is closely related to the physical assumption that the result of counting the microstates of a system of independent particles caged in a line of length *L* does not change if different energy eigenfunctions are chosen. More specifically, it is independent on whether Dirichlet or Neumann boundary conditions are satisfied.

Another natural conjecture concerns the question: does a rational $h<\frac{2}{3}$ exist such that, for $r=o(m^{h})$, then

(43) $$p_{s}\left(r,m\right)∼\frac{\rho_{s}\left(r,m\right)}{r!}\;?$$

Here, we have used the obvious convention that $\rho_{s}\left(\cdot \right)$ and $\rho_{s}^{*}\left(\cdot \right)$ stand for the number of compositions following the choice $\lambda_{k}=k^{2}$. It is straightforward to notice that $r!p_{s}\left(r,m\right)\geq \rho_{s}\left(r,m\right)$, therefore another bound is needed to prove Statement (43). This conjecture is inspired by the validity of the analogous result (10), connecting partitions and compositions with integer values for $r=o(m^{\frac{1}{3}})$.

We conclude this analysis of the partitions generated by the sequence $\lambda_{k}=k^{2}$, k>0, mentioning the asymptotic results of [22]. For the expected number of summands for the unrestricted partition $p_{s}\left(m\right)$, from their Corollary 8-(i),

(44) $$\bar{r}\left(m\right)=\frac{\sum_{r=0}^{\infty }r\;\;p_{s}\left(r,m\right)}{\sum_{r=0}^{\infty }p_{s}\left(r,m\right)}=\frac{1}{p_{s}\left(m\right)}\sum_{r=1}^{m}r\;\;p_{s}\left(r,m\right)=\frac{\zeta \left(2\right)m^{\frac{2}{3}}}{A}+o\left(m^{\frac{2}{3}}\right)\;.$$

We have used the identity $p_{s}\left(r,m\right)=0$, which holds for r=0 and for r>m. This result is entirely consistent with Equation (31), since the distribution f(x) has mean $\zeta \left(2\right)=\frac{\pi^{2}}{6}$. From Corollary 7-(iii) on the second moment of the same variable, defined as,

(45) $$\bar{r^{2}}\left(m\right)≔\frac{1}{p_{s}\left(m\right)}\sum_{r=1}^{m}r^{2}\;\;p_{s}\left(r,m\right),$$

it is then possible to obtain, for the central second moment,

(46) $$\bar{r^{2}}\left(m\right)-\bar{r}{\left(m\right)}^{2}∼\frac{\zeta \left(4\right)m^{\frac{4}{3}}}{A^{2}}\;.$$

Notably, it grows even faster than *m*, in absolute terms. Thus, we see that the square root of the quadratic dispersion is of the same order as the mean. The counting function $p_{s}\left(m\right)$ is therefore indeed dominated by partitions with $O(m^{2/3})$ summands; however, their spread is relatively wide. By dividing the asymptotic identity (46) by $\bar{r}{\left(m\right)}^{2}$, we obtain a constant, i.e., a finite relative fluctuation. This denotes the low-temperature regime expectation for excited states, as we shall see, for a one-dimensional gas.

Until now, we have considered sequences $\Lambda^{+}$, representing single particles’ energy spectra in one dimension. In more than one dimension, the sequences are notoriously more complex and many terms are repeated. We shall not consider two-dimensional models, since they share many similarities with the sequence $\lambda_{k}=k$. In three dimensions, the number of repetitions of each term tend to grow in advancing the sequence, despite there being a finite fraction of absent integers [26]. We shall refer to such sequence simply as $\Sigma^{+}$. It is a sequence of non-decreasing positive integers, whereby the multiplicity of each term in the sequence coincides with the number of its compositions as the sum of three squares. The first terms of the series are

(47) $$\Sigma^{+}=\left\{1,1,1,2,2,2,3,4,4,4,5,5,5,5,5,5,6,6,6,8,8,8,\ldots \right\}\;.$$

To start our survey, we preliminarily mention the following result. The total number of compositions of all integers which are less than or equal to λ, written as the sum of three squares of non-negative integers, is asymptotically given by [26],

(48) $$N\left(\lambda \right)=\sum_{k=1}^{\lambda }\rho_{s}^{*}\left(3,k\right)∼\frac{\pi }{6}\lambda^{\frac{3}{2}}\;.$$

This is the number of terms of the sequence $\Sigma^{+}$ less or equal than λ for large values of λ. In the above example, N(8)=22. Sequences of the family $N\left(\lambda \right)∼b\lambda^{\beta }$, with 1<β<2, were also briefly considered in [37], without all the necessary proofs. For $\beta =\frac{3}{2}$, which interests us here, we have,

(49) $$\log p_{\Sigma^{+}}\left(m\right)∼\frac{5}{3}\;\;B\;\;m^{\frac{3}{5}}\;,$$

(50) $$B={\left[\frac{\pi }{4}\;\Gamma \left(\frac{5}{2}\right)\;\zeta \left(\frac{5}{2}\right)\right]}^{\frac{2}{5}}\;.$$

The authors of [37] also considered $p_{\Sigma^{+}}\left(r,m\right)$, which is essentially $p_{\Sigma^{+}}\left(m\right)$ multiplied by distribution, not given explicitly. However, from [37], it transpires that the argument of such distribution is $r\;\;m^{-\frac{3}{5}}$, multiplied by a constant. That was calculated as the expected number of summands in [22]. They both agree on

(51) $$\bar{r}\left(m\right)∼\frac{2\;\zeta \left(\frac{3}{2}\right)}{3\;\zeta \left(\frac{5}{2}\right)}\;\;B\;\;m^{\frac{3}{5}}\;.$$

One striking result of [22] (Corollary 17) is that the *k*-th moments satisfy

(52) $$\bar{r^{k}}\left(m\right)∼c^{k}m^{\frac{3\;k}{5}}\;,$$

with the constant *c* explicitly given. It follows that, to the leading order,

(53) $$\bar{r^{2}}\left(m\right)-\bar{r}{\left(m\right)}^{2}=o\left(1\right)\;.$$

This is a very sharp result, and it applies to partitions in which the number of summands can be up to *m*. As we shall see, this corresponds to ultra-low temperature regimes, where the mean number of particles in an excited state virtually does not fluctuate. We are not aware of analogous results for the mean number of summands for partitions with a maximum assigned number of summands. If *N* is that maximum, the said number would formally read as

(54) $$\bar{r}\left(N,m\right)=\frac{\sum_{r=1}^{N}r\;\;p_{\Lambda^{+}}\left(r,m\right)}{\sum_{r=1}^{N}p_{\Lambda^{+}}\left(r,m\right)}\;.$$

Finally, we point out that the analogous of the conjecture (43) for the above described sequence $\Lambda^{+}$ entails hypothesizing the existence of $h<\frac{3}{5}$ such that, for $r=o(m^{h})$, then

(55) $$p_{\Sigma^{+}}\left(r,m\right)∼\frac{\rho_{s}\left(3r,m\right)}{r!}\;.$$

To complete our survey, we next report on results concerning the number of compositions of *m* as a sum of an arbitrary number of squares of integers, i.e., $\lambda_{k}=k^{2}$ and k∈Z, or as the sum of squares of non-null integers, i.e., $\lambda_{k}=k^{2}$ and k∈Z\{0}. We shall call them $\tau_{s}^{*}\left(r,m\right)$ and $\tau_{s}\left(r,m\right)$, respectively. These sets differ from the previous sets in that, until now, we had only considered the case of non-negative integers. However, the following simple relation holds:

(56) $$\tau_{s}\left(r,m\right)=2^{r}\rho_{s}\left(r,m\right)\;,$$

which can be understood noticing that each summand $k_{i}^{\;2}$ in $\rho_{s}\left(r,m\right)$ is counted twice in $\tau_{s}\left(r,m\right)$, first as $k_{i}^{\;2}$ and then as ${\left(-k_{i}\right)}^{2}$. For the same reason, the following also holds:

(57) $$\tau_{s}^{*}\left(r,m\right)\leq 2^{r}\rho_{s}^{*}\left(r,m\right)\leq 2^{r}\tau_{s}^{*}\left(r,m\right)\;,$$

or

(58) $$\frac{1}{2^{r}}\;\tau_{s}^{*}\left(r,m\right)\leq \rho_{s}^{*}\left(r,m\right)\leq \tau_{s}^{*}\left(r,m\right)\;,$$

where the first inequality holds since the factor $2^{r}$ produces an overcounting of the null terms, while the second is straightforward.

The inequality (58) is not very informative for generic values of r,m; however, it is useful in entropy calculations, for *r* fixed and asymptotically in *m*. In such case, we have [26]

(59) $$\tau_{s}^{*}\left(r,m\right)∼g_{m}\left(\frac{r}{2}\right)+O\left(m^{r/4}\right)\;,\;\;g_{m}\left(x\right)=\frac{\pi^{x}m^{x-1}}{\Gamma (x)}\;.$$

For sufficiently large values of r≫1, using Stirling’s approximation and dropping the less important terms asymptotic in *m*:

(60) $$\log\tau_{s}^{*}\left(r,m\right)∼\frac{r}{2}\log\frac{2\;\;e\;\;\pi \;\;m}{r}\;.$$

By direct use of the inequalities (58), it follows that in the same range

(61) $$\log\rho_{s}^{*}\left(r,m\right)∼\frac{r}{2}\log\frac{m}{r}+O\left(r\right)\;.$$

We deem that a much more refined result would be available, possibly implying $2^{r}\rho_{s}^{*}\left(r,m\right)∼\tau_{s}^{*}\left(r,m\right)$, but we do not pursue this proof here. It is however a reasonable conjecture, since for very large values of *m*, compositions with O(r) zeros are expected to be in subdominant proportions, leading us to hypothesize,

(62) $$\log\rho_{s}^{*}\left(r,m\right)∼\frac{r}{2}\log\frac{e\;\;\pi \;\;m}{2\;\;r}\;.$$

It is to be noted that the leading term in Equation (59), and therefore (60), does not convey $\tau_{s}^{*}\left(r,m\right)$ uniformly in *r* and *m*. For Equation (60) to be viable, however large, *r* should be considered fixed, and *m* arbitrarily large. In fact, the leading term in *m* of Equation (59) would predict that $\tau_{s}^{*}\left(r,m\right)∼0$ as soon as r≫m≫1. The corrective term $O(m^{r/4})$ signals the presence of other dominant terms. A simple lower bound can be found for $\tau_{s}^{*}\left(r,m\right)$ in that range, by considering the subclass of compositions in which each summand takes only the values 0 or 1, i.e.,

(63) τs*(r,m)≥2mrm,ρs*(r,m)≥rm;r≥m,

(64) $$\log\tau_{s}^{*}\left(r,m\right)≳m\log\frac{2\;r}{m}\;,\;\;\log\rho_{s}^{*}\left(r,m\right)≳m\log\frac{r}{m}\;;\;\;r\gg m\gg 1\;.$$

We now present a loose estimate of $\rho_{s}^{*}\left(m,m\right)$, which is however very straightforward. We specialize for the case ${\left(\frac{m}{2}\right)}^{1/2}\in Z^{+}$, but a generalization is a fairly technical endevour. First, we have the obvious,

(65) $$\rho_{s}^{*}\left(r,m\right)\leq \rho^{*}\left(r,m\right)\;.$$

By virtue of Equation (16), also following the steps (17) or (18),

(66) $$\log\rho_{s}^{*}\left(m,m\right)≲2m\log2\;.$$

Then, we consider the *m* compositions in which one summand is m/2 and the remaining m−1 summands are either 0 or 1. They total up to

mm−1m/2,

so that it also holds that,

(67) $$\log\rho_{s}^{*}\left(m,m\right)≳m\log2+\log m\;.$$

Therefore, for large values of *m*,

(68) $$m\log2≲\log\rho_{s}^{*}\left(m,m\right)≲2m\log2\;.$$

## 5. The Physics of Partitions and Compositions with Squares of Integers in One Dimension

We start by considering the well known problem of a gas of noninteracting identical massive particles (mass μ) with fixed energy in a reflective box, treated as a quasi classical system, in the sense that the statistics is classical, but the states are quantized. Counting the states is possible knowing the energy eigenvalues of the wave functions of *N* particles. In fact, given the hypothesis that the particles do not interact, the eigenfunction of every individual particle is that which solves the problem of a free particle caged in a d-dimensional box (in this section, we specialize for the case of a one-dimensional line, while, in the next section, we shall consider a three-dimensional box of sides *L* and volume $V=L^{3}$). We therefore know the total (fixed) energy of the system as the sum of the energy eigenvalues of all the particles, which are well known [14]. They can also be expressed in terms of momenta for the *n*-th particle. In one dimension, one has:

$$\begin{array}{cccc} \begin{array}{c} \frac{p_{n}^{\;2}}{2\mu }=E_{n}=n^{2}\;\epsilon_{0}\;, \end{array} & \;(69a)\; & \begin{array}{c} \epsilon_{0}=\frac{\hbar^{2}\pi^{2}}{2\mu L^{2}}\;. \end{array} & \;(69b) \end{array}$$

In this work, we shall consider both Dirichlet (70a) and Neumann (70b) boundary conditions:

$$\begin{array}{cccc} \begin{array}{c} n\in Z^{+}\;; \end{array} & \;(70a)\; & \begin{array}{c} n\in Z\;. \end{array} & \;(70b) \end{array}$$

In the classical regime, the proof that the results do not depend on the choice between (c) and (c’) can be made rigorous. Here, we choose Neumann boundary conditions, and it is not a difficult exercise to verify the analogous steps for Dirichlet boundary conditions. In the quantum regime, we shall instead use both the Dirichlet and the Neumann boundary conditions, according to convenience. To be more specific, we shall first interpret the mathematical results on partitions of the previous section in the context of Dirichlet boundary conditions, but the results on the momenta and in three dimensions (see next section), i.e., from Equation(44) to Equation (53) are best interpreted in the context of Neumann boundary conditions. We shall point out, however, when and why in the quantum regime this freedom must be approached with care.

The total energy is the sum over all the *N* particles. Suppose then that such total be given by,

(71) $$E=m\epsilon_{0}\;,\;\;\;m=\frac{2\mu EL^{2}}{\hbar^{2}\pi^{2}}\;,$$

where *E* is the total energy. The number Ω is therefore the number of compositions of *m* into r=N squares of non-negative integers. By considering the compositions of *m*, we are still formally treating the particles as distinguishable. We consider the case m≫N, i.e., standard classical assumptions apply. In fact, the parameter *m* should satisfy $m\gg N^{\frac{3}{2}+x}$, with $x\in R^{+}$ that we have not yet determined. From Equations (60) and (62), after dividing for the usual N! factor to fix the distinguishability issue (and in accordance with the conjecture (43)), the entropy up to order *N* reads

(72) $$S/k_{B}∼\frac{N}{2}\log\frac{e\;\;\mu EL^{2}}{N\pi \;\hbar^{2}}+N-N\log N=N\log\left[\frac{L}{N}{\left(\frac{\mu E}{N\pi \;\hbar^{2}}\right)}^{\frac{1}{2}}\right]+\frac{3N}{2}\;,\;E\gg \frac{\hbar^{2}\pi^{2}N}{2\mu L^{2}}\;.$$

The Gibbs factor is indispensable to regain the L/N term correctly; however, the E/N term is accounted for by counting the compositions. We suspect that some confusion may surround this point, thus we now consider the problem of a monoatomic Bose gas in a one-dimensional square-well. The key to our reasoning resides in part in the validity of the conjecture (43), for sufficiently high energy regimes. We must also assume that $\rho_{s}^{*}\left(r,m\right)≃\rho_{s}\left(r,m\right)$ for large enough *m*.

In the classical (high energy per particle) scenario, we have apparently two main routes. One has been just outlined, leading from Equations (62)–(72). Notice that in one dimension the momenta can be well ordered in a one-to-one match with each particle. As already noticed, in such a situation, the condition of indistinguishability may be approached formally in two ways: either we divide the total number of states by the Gibbs factor, or we sum (or integrate) over a well-ordered sequence of momenta. This is a similar approach to the one that led to the calculation (28), but for an appropriate replacement of the individual energies with the squares of the momenta:

(73) $$V=\frac{\int_{0}^{\sqrt{2\mu E}}dp_{1}0^{\sqrt{2\mu E-p_{1}^{2}}}dp_{2}\cdots \int_{0}^{\sqrt{2\mu E-p_{1}^{2}-\ldots -p_{N-1}^{2}}}dp_{N}\;H\left(p_{1}^{2},p_{2}^{2},\ldots ,p_{N}^{2}\right)}{{\left(2\mu \;\;\epsilon_{0}^{\prime }\right)}^{\;N/2}}\;.$$

This second route leads again to Equation (72).

We stress the importance of the condition $\rho_{s}^{*}\left(r,m\right)≃\rho_{s}\left(r,m\right)$, and especially of the conjecture (43) (in three dimensions, the conjecture (55)). From further mathematical developments, one would indeed obtain the critical value of $E/L^{2}$, for any *N*, at which the ‘classical’ way of counting, aided by division by N!, becomes consistent with the traditional quantum procedure of counting the occupation rates of the energy levels. In other words, a proof of Equations (43) and/or (55) would imply the correctness of the Gibbs factor for sufficiently high energy. Ideally, $\log p_{s}\left(r,m\right)$ would provide the entropy of the noninteracting gas across the entire range, from deepest in the quantum regime to arbitrarily high energies. This constitutes a slight shift of perspective compared to traditional views, which see the entropy as requiring distinct counting procedures.

Once granted the importance of $\log p_{s}\left(r,m\right)$, let us derive some rigorous results. To our knowledge, they have not been pointed out before, in the microcanonical ensemble formalism. For the bosonic gas in a line, r=N, with the usual quantum constraint that

(74) $$p_{n}^{2}=\frac{\hbar^{2}\pi^{2}}{2\mu L^{2}}\;n^{2},\;\;m=\frac{2\mu EL^{2}}{\hbar^{2}\pi^{2}}\;.$$

Here, we assume Dirichlet bounday conditions, i.e., $n\in Z^{+}$. We want to use Equation (42). We point out that the ground state has an energy $E_{0}$ such that,

(75) $$E_{0}=\frac{\hbar^{2}\pi^{2}N}{2\mu L^{2}}\;,$$

(76) $$m-N=\frac{2\mu \left(E-E_{0}\right)L^{2}}{\hbar^{2}\pi^{2}}=\frac{2\mu E^{*}L^{2}}{\hbar^{2}\pi^{2}}\;,$$

where $E^{*}=E-E_{0}$ is the excitation energy.

Via the asymptotic result (30), in close proximity to the ground state, we arrive at:

(77) $$S/k_{B}∼\;3A{\left(\frac{2\;\;\mu \;\;E^{*}L^{2}}{\hbar^{2}\pi^{2}}\right)}^{\frac{1}{3}},$$

(78) $$T={\left(\frac{\partial S}{\partial E^{*}}\right)}^{-1}∼\;{\left(k_{B}A\right)}^{-1}{\left(\frac{\hbar^{2}\pi^{2}E^{*2}}{2\;\;\mu \;\;L^{2}}\right)}^{\frac{1}{3}}\;,\;\;E^{*}∼\frac{\sqrt{2\;\;\mu }\;\;L}{\hbar \pi }\;{\left(A\;\;k_{B}T\right)}^{3/2}\;.$$

An alternative route to the same relation in the critical region exploits the finite difference Equation (35), with $E=m\epsilon_{0}$ and the definition (69b) intended, which we propose in the weaker form,

(79) $$p_{s}\left(N,m\right)-p_{s}\left(N,m-1\right)∼Am^{-\frac{2}{3}}p_{s}\left(N,m\right)\;.$$

By taking,

(80) $$\frac{1}{T}=\frac{S(N,m)-S(N,m-1)}{\epsilon_{0}}\;,$$

we get

(81) $$\frac{\epsilon_{0}}{k_{B}T}=\log\frac{p_{s}\left(N,m\right)}{p_{s}\left(N,m-1\right)}≃-\log\left(1-Am^{-\frac{2}{3}}\right)\;,$$

(82) $$m^{\frac{2}{3}}=A{\left[1-\exp\left(-\frac{\epsilon_{0}}{k_{B}T}\right)\right]}^{-1}.$$

One can verify that this expression is quite inaccurate in the zero temperature limit, giving,

(83) $$E\left(T∼0^{+}\right)=A^{\frac{3}{2}}\epsilon_{0}\;,$$

which is the counterpart to the inaccurate Equation (34). Notice that the difference Equation (80) has little meaning very close to the ground state.

For larger temperatures, we obtain,

(84) $$E=\epsilon_{0}^{-\frac{1}{2}}\;{\left(A\;\;k_{B}T\right)}^{\frac{3}{2}}\;,$$

which confronts favourably with the result (78), since we are exploring the region $N=O(m^{2/3})$ and therefore $E∼E^{*}$.

Equation (78) lacks an explicit dependence on *N*, which is mathematically an open question. However, such dependence is very weak, for large *N* and down to $N=O(m^{2/3})$ (see the discussion leading up to and following Equation (31)). A sharp decline of the rate of increase of entropy with energy is encountered, as soon as the number of particles (the summands) becomes less than ‘typical’ for that value of the integer *m*. From both Equations (31) and (44), this critical point satisfies

(85) $$\frac{2\;\;\mu \;\;E_{c}\;\;L^{2}}{\hbar^{2}\pi^{2}}{\left[\frac{\zeta (2)}{A}\right]}^{\frac{3}{2}}=N^{\frac{3}{2}},\;\;E_{c}=\frac{\hbar^{2}\pi^{2}\;\;N^{3/2}}{2\;\;\mu \;\;L^{2}}{\left[\frac{A}{\zeta (2)}\right]}^{\frac{3}{2}}=E_{c}^{*}+\frac{\hbar^{2}\;\;\pi^{2}\;\;N}{2\;\;\mu \;\;L^{2}}≃E_{c}^{*}\;;$$

or roughly

(86) $$k_{B}T_{c}≃\frac{\hbar^{2}\;\;\pi^{2}\;\;N}{2\;\;\zeta \left(2\right)\;\;\mu \;\;L^{2}}=\frac{3\;\;\hbar^{2}\;\;N}{\mu \;\;L^{2}}\;.$$

In one dimension, there is no phase transition [14]; however, $T_{c}$ marks an approximate temperature at which a macroscopic number of particles occupies the ground state. The mathematical explanation is that, well below $T_{c}$, the number of particles is so large as to be redundant, with respect to the ‘optimal’ number for the available partitions of the total energy. Therefore, many particles can occupy the ground state almost ‘for free’. However, from the estimate (86), it transpires that, differently from higher dimensions, $T_{c}$ approaches zero when the size of the system, *L*, and *N* approach infinity with equal rates.

Also notice that, below $T_{c}$, the probability that $n_{0}$ particles occupy the ground state is $\frac{p_{\sigma }\left(N-n_{0},m-N\right)}{p{*}_{\sigma }\left(N,m-N\right)}=\frac{p_{\sigma }\left(N-n_{0},m-N\right)}{p{*}_{s}\left(N,m\right)}$, which is asymptotically proportional to the ratio $\frac{p_{\sigma }\left(N-n_{0},m-N\right)}{p_{s}\left(m-N\right)}$. From the asymptotic form (40), this quantity is approximately proportional to the function *g*, and therefore it has a maximum when $N-n_{0}=O\left[{\left(m-N\right)}^{2/3}\right]$, with m−N the units of excitation energy $E^{*}/\epsilon_{0}$ (see [4] for an analogous result for one-dimensional harmonic traps, which also applies to two-dimensional ideal Bose gases).

We now deepen three related questions: how to characterize the region closely around $T_{c}$, which states the particles occupy below $T_{c}$; finally, what are the typical number fluctuations deeper into the quantum regime.

To get a flavor of the dependence on *N* at the boundary of the critical region $N=O(m^{2/3})$, i.e., around $T_{c}$, we observe that the function *f* can be simplified for either small or large arguments. The entropy is taken from Equations (30) and (31), with the rightmost term below bearing the *N*-dependence,

(87) $$S/k_{B}=\log p_{s}\left(N,m\right)∼\;3Am^{\frac{1}{3}}-\frac{2}{3}\log m+\log\left(A\right)+\log f(N\;\;A\;\;m^{-\frac{2}{3}})\;.$$

When the argument of *f* is large (but finite), i.e., just below $T_{c}$, we inherit the expression of Equation (34), and by deriving with respect to *E*,

(88) $$\frac{\epsilon_{0}}{k_{B}T}∼\frac{3\;\;Am^{\frac{1}{3}}-2+2A\;\;Nm^{-\frac{2}{3}}}{3m}≃Am^{-\frac{2}{3}}\left(1+\frac{2}{3}\frac{N}{m}\right)\;.$$

Since N/m is small,

(89) $$A^{3}{\left(\frac{k_{B}T}{\epsilon_{0}}\right)}^{3}∼m\left(m-2N\right)\;,$$

which corrects Equations (78) and (84) to

(90) $$E∼\frac{\sqrt{2\;\;\mu }\;L}{\hbar \pi }\;{\left(A\;\;k_{B}T\right)}^{3/2}+\frac{\hbar^{2}\pi^{2}\;\;N}{2\;\;\mu \;\;L^{2}}+\frac{1}{2}\frac{\hbar^{5}\pi^{5}}{{\left(2\;\;\mu \right)}^{5/2}\;\;L^{5}}\frac{N^{2}}{{\left(AkT\right)}^{3/2}}=E^{*}+E_{0}+\mathrm{corr}\;,$$

$$\mathrm{if}\;N∼\gamma {\left(\frac{2\;\;\mu \;\;L^{2}\;\;E}{\hbar^{2}\pi^{2}}\right)}^{2/3}\;\mathrm{and}\;\gamma \;\mathrm{large}\;\mathrm{but}\;\mathrm{finite}.$$

Notice that the ground state energy has emerged quite naturally, and it is the first signature imprinted by *N*, while the corrective term is short-lived and should not be considered at very low temperatures.

At the other end of the boundary of the region $N=O(m^{2/3})$, i.e., just above $T_{c}$, we can use [37] $\log f(x)∼-\frac{\pi^{2}}{4x}\;\;\;\left(x∼0^{+}\right)$, and for the entropy and its energy derivative,

(91) $$S/k_{B}=\log p_{s}\left(N,m\right)∼\;3Am^{\frac{1}{3}}-\frac{2}{3}\log m+\log\left(A\right)-\frac{\pi^{2}\;\;m^{\frac{2}{3}}}{4NA}\;,$$

(92) $$\frac{\epsilon_{0}}{k_{B}T}∼\frac{3\;\;Am^{\frac{1}{3}}-2}{3m}-\frac{\pi^{2}}{6ANm^{\frac{1}{3}}}≃Am^{-\frac{2}{3}}\left(1-\frac{\pi^{2}}{6A}\frac{m^{\frac{1}{3}}}{N}\right)\;,$$

which now corrects Equations (78) and (84) to

(93) $$E∼\frac{\sqrt{2\;\;\mu }\;L}{\hbar \pi }\;{\left(A\;\;k_{B}T\right)}^{3/2}-\frac{k_{B}T\;\;\pi^{2}}{4\;\;A\;\;N}\;,$$

$$\mathrm{if}\;N∼\gamma {\left(\frac{2\;\;\mu \;\;L^{2}\;\;E}{\hbar^{2}\pi^{2}}\right)}^{2/3}\;\mathrm{and}\;\gamma \;\mathrm{is}\;\mathrm{small}\;\mathrm{but}\;\mathrm{finite}.$$

Eventually, the negative correction in the energy’s asymptotic expression (93) will balance the positive leading term for some small (compared to *E*) *N*, but the emerging functional form E(N,L,T) is not known from combinatorics theorems. Ultimately in the classical limit, *E* will become proportional to *T*. By first derivation of both estimates (90) and (93) with respect to *T*, one can easily obtain the specific heat. In both cases, the term proportional to $\sqrt{T}$ is the dominant term and there is no change of convexity (absence of a transition).

Below $T_{c}$, $E^{*}<E_{c}^{*}$, and the excitation energy must be shared by the ‘typical’ number of summands of the unrestricted partitions counted by $p_{\sigma }\left(m-N\right)$ (recall that $p_{\sigma }$ shares the same astmptotics of $p_{s}$). This typical number is the mean number of particles in an excited state, which we call $\bar{N^{*}}$. Therefore, the following must hold, at least approximately,

(94) $$\bar{N^{*}}≃\frac{\zeta (2)}{A}{\left[\frac{2\;\;\mu \;\;E^{*}\;\;L^{2}}{\hbar^{2}\pi^{2}}\right]}^{\frac{2}{3}}≃\frac{NT}{T_{c}}\;.$$

Therefore, when $T<T_{c}$, the fraction of particles in excited states is circa $\frac{\bar{N^{*}}}{N}≃\frac{T}{T_{c}}$ and $1-\frac{T}{T_{c}}$ the fraction of particles in the ground state.

As we have said, Equations (44)–(46) are more effectively interpreted assuming Neumann boundary conditions, i.e., the ground state has energy zero and $\epsilon_{0}$ is the energy of the first excited state. In order for the definition (44) to be rigorously applicabile, after the identification $r=N^{*}$ and $m=m^{*}$ (the units of excitation energy), we must assume that $N\geq m^{*}$ holds, since otherwise the upper limit of the sums should be *N* (in which case, we lack rigorous mathematical results). In other words, Equations (44)–(46) are exact predictions for $\bar{N^{*}}$ if $N\geq m^{*}$, and are only approximately valid in the region for *N* between $m^{*2/3}$ and $m^{*}$. Therefore, the result (46) implies that

(95) $$\bar{N^{*2}}-{\bar{N^{*}}}^{2}∼\frac{\zeta (4)}{A^{2}}{\left(\frac{2\;\;\mu \;\;L^{2}E^{*}}{\hbar^{2}\;\;\pi^{2}}\right)}^{\frac{4}{3}}∼\zeta \left(4\right)\;{\left(\frac{2\;\;\mu \;\;L^{2}\;\;k_{B}T}{\hbar^{2}\;\;\pi^{2}}\right)}^{2}=\frac{\zeta (4)}{\zeta {\left(2\right)}^{2}}\;\frac{N^{2}\;T^{2}}{T_{c}^{2}}\;,$$

which is rigorous if $E^{*}\leq E_{0}=O\left(\frac{E_{c}^{*}}{\sqrt{N}}\right)$, or $T\leq O\left(\frac{T_{c}}{N^{1/3}}\right)$. After dividing this quantity by ${\bar{N^{*}}}^{2}$, we find that the relative fluctuations are constant. This result is rigorously valid deep into the quantum regime. However, it may still hold approximately, somewhat closer to $T_{c}$, although further investigations would be needed. In summary,

(96) $$\frac{\bar{N^{*2}}-{\bar{N^{*}}}^{2}}{{\bar{N^{*}}}^{2}}∼\frac{\zeta (4)}{\zeta {\left(2\right)}^{2}}\;.$$

It is informative that, since $n_{0}=N-N^{*}$, where $n_{0}$ is the number of particles in the ground state, and, since *N* is fixed, then Equation (95) also immediately defines $\bar{n_{0}^{2}}-{\bar{n_{0}}}^{2}$. However, for ${\bar{n_{0}}}^{2},$ we have,

(97) $${\bar{n_{0}}}^{2}={\bar{N-N^{*}}}^{\;2}=N^{2}{\left(1-\frac{T}{T_{c}}\right)}^{2}\;,$$

and, consequently,

(98) $$\frac{\bar{n_{o}^{2}}-{\bar{n_{0}}}^{2}}{{\bar{n_{0}}}^{2}}∼\frac{\zeta (4)}{\zeta {\left(2\right)}^{2}}\;\frac{T^{2}}{{\left(T_{c}-T\right)}^{2}}\;.$$

Well inside the quantum regime, below $E_{0}$, where the mesoscopic system presents overpopulation of the ground state, the above equation is rigorous and the relative fluctuations of the ground state tend to vanish. Approaching $T_{c}$, granted the validity of the above equations, those fluctuations tend to grow more than linearly in temperature, although their apparent divergence is possibly an artefact of our assumptions. More specifically, although weak in the range between $E_{0}$ and $E_{c}$, we have altogether omitted the precise dependence of the partitions on the number of summands. This issue may be addressed by resolving the mathematical open problem stated in Equation (54).

We mention that the freedom of choice of boundary conditions should be approached critically. The results of [37], insofar as they barely discriminate between two different single-particle spectra that share the same asymptotic N(λ), are very powerful results. This is clear in the steps from Equations (38)–(42). Thus, the inclusion or exclusion of the zero from the energy eigenvalues does not change the emerging physics, since it just results in a shift of one place in the sequence Λ. In mesoscopic systems, this should not be taken entirely for granted. There certainly exists a region of parameters space where surface effects might become important [14]. At that point, the physics at the boundary should be thoroughly considered. In the language of partitions, this entails that *m* is not yet large enough to lie in the asymptotic regime.

We have seen that the problem of entropy of a one-dimensional boson gas can be reduced to counting how many ways an integer *m* can be written as the sum of non decreasing squares of positive integers. For each choice of *m*, there is an optimal number of summands (the particles) around the region $N=O(m^{2/3})$, to which it corresponds a critical temperature. Therefore, the entropy is ‘large’ and almost independent of the particles, when the particles exceed that number. For smaller values of *N*, there is an abrupt decrease in entropy because *N* becomes a limiting factor for the number of partitions. For larger values, we enter deep into a quantum regime, whose fluctuations of excitation numbers have been characterized.

## 6. The Three-Dimensional Boson Gas and Open Problems

We first briefly consider a classical gas of noninteracting identical massive particles inside a reflective cubic box of side *L* (volume $V=L^{3}$), with fixed energy. The energy eigenvalues are now expressed as

$$\begin{array}{cccc} \begin{array}{c} \frac{p_{n_{x},n_{y},n_{z}}^{\;2}}{2\mu }=E_{n_{x},n_{y},n_{z}}=\left(n_{x}^{2}+n_{y}^{2}+n_{z}^{2}\right)\;\epsilon_{0}\;, \end{array} & \;(99a)\; & \begin{array}{c} \epsilon_{0}=\frac{\hbar^{2}\pi^{2}}{2\mu L^{2}}\;. \end{array} & \;(99b) \end{array}$$

The Dirichlet (100a) and Neumann (100b) boundary conditions are spelled out as:

$$\begin{array}{cccc} \begin{array}{c} n_{x},n_{y},n_{z}\in Z^{+}\;; \end{array} & \;(100a)\; & \begin{array}{c} n_{x},n_{y},n_{z}\in Z\;. \end{array} & \;(100b) \end{array}$$

In this section, only the Neumann boundary conditions will be considered. Thus, rather than using the definitions (71), it is more practical to define *m* directly in terms of the excitation energy $E^{*}$, since the zero of the energy now coincides with the global ground-state energy. We thus define

(101) $$E^{*}=m\epsilon_{0}\;,\;\;\;m=\frac{2\mu E^{*}L^{2}}{\hbar^{2}\pi^{2}}\;.$$

The number Ω is now the number of compositions of *m* into r=3N squares of non negative integers, with m≫3N. In fact, the parameter *m* should satisfy $m\gg N^{\frac{5}{3}+y}$, with $y\in R^{+}$ that we have not yet determined. Similarly to the one-dimensional case, we can use Equations (60) and (62). One must then divide the composition number by N! and substitute $V=L^{3}$, which leads us to the following entropy up to order *N*,

(102) $$S/k_{B}∼\frac{3N}{2}\log\frac{e\;\;\mu EL^{2}}{3N\pi \;\hbar^{2}}+N-N\log N=N\log\left[\frac{V}{N}{\left(\frac{\mu E}{3N\pi \;\hbar^{2}}\right)}^{\frac{3}{2}}\right]+\frac{5N}{2}\;,\;E\gg \frac{\hbar^{2}\pi^{2}N}{2\mu L^{2}}\;,$$

which is the well-known Sackur–Tetrode equation [13], here obtained via the counting of integer quantities.

The Gibbs factor now fixes the V/N term correctly, again, with the E/N term accounted for by counting the compositions. Division by N! might not be regarded as ad hoc assumption, should the conjecture (55) be proved correct.

The single-particle spectrum of excitation energy is given by the sequence $\Sigma^{+}$ of Section 4, if each term is multiplied by $\epsilon_{0}$. The quantity Ω is given by $p_{\Sigma^{+}}^{*}\left(N,m\right)$, the number of partitions on $\Sigma^{+}$ with up to *N* summands. Clearly, $p_{\Sigma^{+}}^{*}\left(m,m\right)=p_{\Sigma^{+}}^{*}\left(m\right)$. In the low energy/temperature quantum regime, i.e., *m* not too much larger than *N*, the partitions $p_{\Sigma^{+}}\left(N,m\right)$ cannot be described by the guess (55), since it is incompatible with the correct asymptotics, Equation (49). When N≥m, up to *m* particles (but no more) can occupy an excited state, and the rest N−m particles must be in their ground state (the triplet (0,0,0)). In this situation, the asymptotic result (49) holds rigorously. Furthermore, the mean number $N^{*}$ of excited particles is given by Equation (51). From [37], but also in accordance with [22], first, we have

(103) $$S/k_{B}∼\frac{5}{3}\;\;B\;\;{\left(\frac{2\;\;\mu \;\;L^{2}\;\;E^{*}}{\hbar^{2}\pi^{2}}\right)}^{\frac{3}{5}}\;,$$

and, therefore,

(104) $$k_{B}T∼\frac{E^{*\frac{2}{5}}}{B}{\left(\frac{\hbar^{2}\pi^{2}}{2\;\;\mu \;\;L^{2}}\right)}^{\frac{3}{5}}\;;\;\;E^{*}∼{\left(\frac{2\;\;\mu \;\;L^{2}}{\hbar^{2}\pi^{2}}\right)}^{\frac{3}{2}}{\left(B\;\;k_{B}\;\;T\right)}^{\frac{5}{2}}=\frac{\Gamma \left(\frac{5}{2}\right)\;\zeta \left(\frac{5}{2}\right)\;\;\mu^{\frac{3}{2}}V}{\sqrt{2}\;\hbar^{3}\pi^{2}}\;\;{\left(k_{B}T\right)}^{\frac{5}{2}}\;.$$

The latter formula is standard from quantum statistical mechanics [14].

Next, we find that $p_{\Sigma^{+}}^{*}\left(N,m\right)$ is still very well approximated by $p_{\Sigma^{+}}^{*}\left(m\right)$ for values of *N* that are larger than $O(m^{\frac{3}{5}})$. This is the condensed phase, with an extremely weak dependence on *N*. Once that threshold is crossed, *m* cannot be partitioned any more into its optimal number of summands, and a marked dependence on *N* for $p_{\Sigma^{+}}^{*}\left(N,m\right)$ develops. The condensed phase disappears. From the functional relation (51), we find the optimal number of summands in correspondence with its associated critical excitation energy $E_{c}^{*}$,

(105) $$\frac{2\;\;\mu \;\;E_{c}^{*}\;\;L^{2}}{\hbar^{2}\pi^{2}}{\left[\frac{2\;B\;\zeta \left(\frac{3}{2}\right)}{3\;\zeta \left(\frac{5}{2}\right)}\right]}^{\frac{5}{3}}=N^{\frac{5}{3}},\;\;E_{c}^{*}=\frac{\hbar^{2}\pi^{2}\;\;N^{5/3}}{2\;\;\mu \;\;L^{2}}{\left[\frac{3\;\zeta \left(\frac{5}{2}\right)}{2\;B\;\zeta \left(\frac{3}{2}\right)}\right]}^{\frac{5}{3}}\;,$$

and via the relations (104), the temperature $T_{c}$:

(106) $$k_{B}T_{c}=\frac{2\;\;\pi \;\;\hbar^{2}}{\mu }{\left[\frac{N}{\zeta \left(\frac{3}{2}\right)V}\right]}^{\frac{2}{3}}\;.$$

Unlike the one-dimensional case, this temperature is finite in the thermodynamic (bulk) limit, V/N→const.

For lower temperatures, the latter formula can be inverted to give the average number of particles in the excited state $\bar{N^{*}}$, as a function of temperature (see also previous section). According to the definition (51), the formula is exact for N≥m, but it is a valid approximation up to the critical point. Thus,

(107) $$\frac{\bar{N^{*}}}{N}≃{\left(\frac{T}{T_{c}}\right)}^{\frac{3}{2}}.$$

Arguing similarly to the one-dimensional case, we also find that, below $T_{c}$, the conditional probability that the excitation energy is $E^{*}=m\epsilon_{0}$ when $N_{0}$ particles occupy the ground state has a maximum for $N-N_{0}=O\left(m^{\frac{3}{5}}\right)$.

The result (53) informs us that, below $T_{c}$,

(108) $$\bar{N^{*2}}-{\bar{N^{*}}}^{2}≃0\;,$$

where the equality holds rigorously for N≥m, or

$$k_{B}T\leq \frac{\hbar^{2}\pi^{2}}{2\;\;B\;\;\mu \;\;L^{2}}\;N^{\frac{2}{5}}.$$

The number of particles occupying the ground state is $N-\bar{N^{*}}$, so it also has no fluctuations well below the critical point. This result contradicts analogous results in the grand canonical ensemble, which predicts non-vanishing fluctuations, but it confirms the findings of the canonical ensemble [14].

We lack a detailed description at the wings of the critical regions of condensation. A much richer characterization of the quantities $p_{\Sigma^{+}}\left(N,m\right)$ and $p_{\Sigma^{+}}^{*}\left(N,m\right)$ would be needed, especially for detailing the *N* dependence accurately. This may turn crucial, following the mentioned remarks in [15] concerning the mathematical foundations of the grand canonical ensemble and the possibility to find different results in the microcanonical ensemble.

## 7. Some References to the Grand Canonical Ensemble

We shall consider mainly the one-dimensional model in the grand canonical ensemble, mentioning some results in three dimensions. We recall the following way to link the theory of partitions, defining the microcanonical ensemble approach, to the grand canonical ensemble, in the case of non-interacting identical bosons. The grand canonical partition is a function $Z_{G}=Z_{G}\left(\mu_{c},V,T\right)$, where $\mu_{c}$ is the chemical potential. For practicality, we shall use instead the negative of the chemical potential, $\chi =-\mu_{c}$. Set ν and η such that $\chi =-k_{B}T\log\nu$ and $\epsilon_{0}=-k_{B}T\log\eta$. Then,

(109) $$Z_{G}=g\left(\nu ,\eta \right)\;,$$

where g(x,z) is defined in Equation (29). It follows from the definitions that

(110) $$Z_{G}=\sum_{n=0}^{\infty }\sum_{m=0}^{\infty }\exp\left[-\frac{n\;\chi +m\;\epsilon_{0}}{k_{B}T}+\frac{S(n,V,m\;\epsilon_{0})}{k_{B}}\right]\;,$$

and under the assumption $L^{d}=O\left(\bar{N}\right)$, $E=O(\bar{N})$, $\bar{N}\to \infty$ the thermodynamics do not differ from the microcanonical ensemble. More explicitly, the thermodynamic potential *G* is $G=-k_{B}T\log Z_{G}$, therefore the product decomposition in (29) is useful. In the one-dimensional case with $\lambda_{k}=k^{2}$, in fact,

(111) $$G=k_{B}T\sum_{k=1}^{\infty }\log\left(1-\nu \eta^{\lambda_{k}}\right)=k_{B}T\sum_{k=1}^{\infty }\log\left(1-\nu \;\;\eta^{k^{2}}\right)=k_{B}T\sum_{k=1}^{\infty }\log\left(1-e^{\;-\left(\chi +k^{2}\epsilon_{0}\right)/\left(k_{B}T\right)}\right)\;,$$

for Dirichlet boundary conditions, and

(112) $$G=k_{B}T\sum_{k=0}^{\infty }\log\left(1-\nu \eta^{\lambda_{k}}\right)=k_{B}T\sum_{k=0}^{\infty }\log\left(1-\nu \;\;\eta^{k^{2}}\right)=k_{B}T\sum_{k=0}^{\infty }\log\left(1-e^{\;-\left(\chi +k^{2}\epsilon_{0}\right)/\left(k_{B}T\right)}\right)\;,$$

for Neumann boundary conditions. We assume the latter, in which case χ>0 must hold.

Average thermodynamics quantities can be obtained through proper derivations. The mean occupation number of the *k*-th state with energy $\epsilon_{k}=\lambda_{k}\;\epsilon_{0}$ is given by $\bar{n_{k}}=-\frac{\partial G}{\partial \epsilon_{k}}$; therefore,

(113) $$\bar{n_{k}}={\left(\nu^{-1}\eta^{-k^{2}}-1\right)}^{-1}={\left(e^{\;\left(k^{2}\epsilon_{0}+\chi \right)/\left(k_{B}T\right)}-1\right)}^{-1}\;.$$

The average excitation energy can be computed more directly from $E=\sum \epsilon_{k}\bar{n_{k}}$,

(114) $$E^{*}=\epsilon_{0}\sum_{k=0}^{\infty }\frac{k^{2}}{e^{\;\left(k^{2}\epsilon_{0}+\chi \right)/\left(k_{B}T\right)}-1}\;.$$

This sum is usually approximated by an integral (see [15] for a critical analysis of this step),

(115) $$E^{*}≃\epsilon_{0}\int_{0}^{\infty }\frac{z^{2}\;dz}{e^{\;\left(z^{2}\epsilon_{0}+\chi \right)/\left(k_{B}T\right)}-1}=\frac{1}{2}\frac{{\left(k_{B}T\right)}^{\frac{3}{2}}}{\sqrt{\epsilon_{0}}}\int_{0}^{\infty }\frac{z^{\frac{1}{2}}dz}{e^{\chi /(k_{B}T)+z}-1}=\frac{1}{2}\frac{{\left(k_{B}T\right)}^{\frac{3}{2}}}{\sqrt{\epsilon_{0}}}\;\Gamma \left(\frac{3}{2}\right)\;g_{\frac{3}{2}}\left(\frac{\chi }{k_{B}T}\right)\;,$$

with $g_{n}\left(x\right)$ the so-called Bose function of order *n*,

$$g_{n}\left(x\right)=\frac{1}{\Gamma (n)}\int_{0}^{\infty }\frac{z^{\;n-1}\;dz}{e^{\;x+z}-1}=\sum_{k=1}^{\infty }\frac{e^{-kx}}{k^{\;n}}.$$

In the low temperature regime, $\chi ∼0^{+}$, and, since $g_{3/2}\left(0\right)=\zeta \left(3/2\right)$, then at low temperatures,

(116) $$E^{*}∼\frac{1}{2}\Gamma \left(\frac{3}{2}\right)\;\zeta \left(\frac{3}{2}\right)\frac{{\left(k_{B}T\right)}^{\frac{3}{2}}}{\sqrt{\epsilon_{0}}}\;,$$

in accordance with Equation (78).

The average number of particles in the ground state, $\bar{n_{0}}$, and in the excited state, $\bar{N^{*}}$, are respectively given by,

(117) $$\bar{n_{0}}={\left(e^{\chi /(k_{B}T)}-1\right)}^{-1}=\frac{\nu }{1-\nu }\;,$$

(118) $$\bar{N^{*}}=\bar{N}-\bar{n_{0}}=\sum_{k=1}^{\infty }\frac{1}{e^{\;\left(k^{2}\epsilon_{0}+\chi \right)/\left(k_{B}T\right)}-1}≃\frac{1}{2}\;\Gamma \left(\frac{1}{2}\right)\;g_{\frac{1}{2}}\left(\frac{\chi }{k_{B}T}\right){\left(\frac{k_{B}T}{\epsilon_{0}}\;\right)}^{\frac{1}{2}}.$$

Dividing the latter by *L*, and recalling the definition of $\epsilon_{0}$,

(119) $$\frac{\bar{N^{*}}}{L}≃\frac{1}{\hbar }\;g_{\frac{1}{2}}\left(\frac{\chi }{k_{B}T}\right){\left(\frac{\mu \;\;k_{B}T}{2\;\;\pi }\right)}^{\frac{1}{2}}\;.$$

We look for solutions in the thermodynamic limit, therefore for a finite ratio $\bar{N^{*}}/L$. Since $g_{1/2}$ is monotonic and unbounded, then χ exists that solves the equation for any finite $\bar{N^{*}}/L$, at any temperature. Decreasing *T*, $g_{1/2}$ must approach a large number. Since $g_{\frac{1}{2}}\left(x\right)$ grows as $\Gamma \left(\frac{1}{2}\right)x^{-1/2}$ for small *x*, then, at a low temperature, χ must be much smaller than $k_{B}T$, and, more concisely,

(120) $$\frac{\bar{N^{*}}}{L}∼\frac{\sqrt{\mu }}{\hbar \;\;\sqrt{2}}\;\frac{k_{B}T}{\sqrt{\chi }}\;.$$

From the identity (117), we also have

(121) $$\frac{\chi }{k_{B}T}=\log\left(1+\bar{n_{0}}\right)-\log\bar{n_{0}}=\log\left(1+\frac{1}{\bar{n_{0}}}\right)\;,$$

which in the low temperature regime gives

(122) $$\bar{n_{0}}≃\frac{k_{B}T}{\chi }∼\frac{\bar{N^{*}}}{L}\;\frac{\hbar \;\;\sqrt{2}}{\sqrt{\mu \;\chi }}\;.$$

By division over *L*, Equation (118) can thus be recast,

(123) $$\frac{N}{L}∼\frac{\bar{N^{*}}}{L}\left(1+\frac{\hbar }{L}\sqrt{\frac{2}{\mu \;\chi }}\right)\;,$$

which shows the absence of condensation when L→∞ at any finite χ (and therefore *T*).

For finite size, there is a critical temperature $T_{c}$ at which $\bar{n_{0}}$ becomes of the order of $\bar{N^{*}}$, through the expression (120), which is simply obtained by equating in (123)

$$\frac{\hbar }{L}\sqrt{\frac{2}{\mu \;\chi }}=1\;,$$

i.e., via relation (120),

(124) $$k_{B}T_{c}≃\frac{2\;\;\hbar^{2}\;\;\bar{N^{*}}}{\mu \;\;L^{2}},$$

which is in some agreement with the estimate (86). We shall further comment on the region below $T_{c}$.

In the grand canonical ensemble, the relative fluctuations in all dimensions obey [13,14]

(125) $$\frac{\bar{n_{k}^{2}}-{\bar{n_{k}}}^{\;2}}{{\bar{n_{k}}}^{\;2}}=1+\frac{1}{\bar{n_{k}}}\;.$$

Thus, they are large for states scarcely populated. Less obvious, even well within the condensed phase, when $\bar{n_{0}}$ is very large, the relative fluctuation of the ground state are still of order unity. Our results from (94) to (98) offer a much richer phenomenology for the one-dimensional mesoscopic gas in the microcanonical approach. At ultra low temperatures, where the formulas given are exact, the relative fluctuations of the ground state tend to vanish quadratically in *T*, where the putative condensed state (not a phase) is firmly established. However, fluctuations grow markedly when the temperature approaches the critical value. This is also the region where the approximations adopted are no longer valid. As we have commented, the divergence may be an artefact of the missing subdominant terms, but the initial growth of the fluctuations may have been correctly captured.

In three dimensions, we have seen that the microcanonical approach offer different results from the grand canonical, apart from the general results from (104) to (107). From Equation (108), we see that the fluctuations of the ground state occupancy, which in absolute terms are given by the same formula, vanish. This is an exact result in the ultra-low temperature region, but it is also approximately verified when approaching the critical temperature from below. Again, the mathematical analysis is incomplete to describe what happens very near the critical region. It is not a novelty that the limits of the grand canonical ensemble in properly capturing the fluctuations of a finite system are discussed [4,5,6,7,8,13,14].

## 8. Conclusions

The mathematics of partitions and compositions are notoriously rich. Even trivially formulated questions have found often non-trivial, sometimes limited answers. Concurrently, microcanonical ensemble calculations in statistical mechanics are comparatively resilient to full exact solutions, even in the simple case of non-interacting particles. These difficulties have led most physicists to often prefer other ensembles, since the asymptotic limits have been implicitly executed beforehand, and in a physically consistent manner. For non-interacting particles, the grand canonical ensemble focuses the computational burden solely on the single-particle energy spectra. Nevertheless, mathematicians do have made significant and insightful progress in partitions theory over several decades, which are not always encompassed in the traditional overviews of statistical mechanics. There are exceptions [34,35,36], and in fact a growing interest has accompanied the need to describe mesoscopic quantum systems within microcanonical ensemble theory [4,5,6,7,8]. Following those steps, we have tried to exploit a small but significant subset of results in partition theory [22,37] to incorporate them in statistical mechanics. More explicitly, we have considered the case of a mesoscopic one-dimensional system, constituted by non-interacting bosons. To give an encompassing picture, we have considered also classical limits, while pointing out the partial lack of rigorous mathematical results applicable in the intermediate regions. We have characterized the behavior of the one-dimensional (mesoscopic) Bose gas behavior around the critical temperature, which marks the cross-over to a condensed region, even without a phase transition. We have found that at ultra-low temperatures, the relative fluctuations of the growing ground state occupation number vanish, which is more akin to canonical ensemble predictions than to grand canonical ensemble predictions. Approaching the critical temperature from below, those fluctuations grow markedly, possibly only apparently without bound (due to the limits of applicability of the mathematical theory). The apparent divergence of the relative fluctuations at $T_{c}$ of Equation (98) may be explained with the extrapolation of a formula, which is only rigorously valid at lower temperatures. The solution of the open problem stated in Equation (54) might clarify this point in the future.

In three dimensions, after recovering correctly the bulk limits at and below the condensation temperature, we have found that in the condensed region the microcanonical ensemble predicts null fluctuations of the ground state occupation number, again much alike in the canonical ensemble theory and in contrast to grand canonical ensemble predictions. In that case, the result seems to extend even on approaching the critical temperature from below. Other results in the thermodynamic (bulk) limit conform to the predictions of the grand canonical ensemble.

Beyond such significant results, albeit not yet fully matching the simplicity and breadth of applicability provided by calculations in the grand canonical ensemble, we have covered a wider range of parameters space to complete our survey. This part could stimulate more interest.

How would one guess the number of unrestricted partitions of *m* into compositions of *d* integer squares? Trusting the extensivity of the microcanonical entropy, guessing that, for free bosonic particles $S/k_{B}\propto {\left(E\;L^{2}\right)}^{\alpha }$, and since $V=L^{d}$, then $\alpha ={\left(1+2/d\right)}^{-1}$ in order for *S* to grow as either *E* or *V* in the thermodynamic limit. The lack of explicit dependence on *N* echoes that $S/k_{B}$ must be approximately the logarithm of the unrestricted partitions of $m\propto E\;L^{2}$ into squares of integers. Thus, $\log p_{s}^{\left(d\right)}\left(m\right)∼m^{\frac{d}{2+d}}$. This crude reasoning could be formally enriched, assuming that the available results in the canonical and grand canonical ensemble are valid also (in the bulk limit) in the microcanonical ensemble. Thus, the average of particles in the excited state and the energy vs. temperature relations could be framed to reconstruct the number of unrestricted partitions and the expected number of summands in those partitions. These mathematical results are obtained with very sophisticated calculations, which would often go beyond the immediate interest of physicists, but familiarity with standard statistical mechanics results could provide a good key for their interpretation.

Another key mathematical question is the characterization of the entropy across the full spectrum of values of *N*. The results available are somewhat narrow. Furthermore, while the asymptotic idenity (10) relates partitions and compositions with integer values, the analogous guesses (43) and (55) are open problems. Additionally, there seems to be little interest in the mathematical community about the problem of counting the partitions involving *r* squares, adding up to any integer number ≤m. The distinction between adding up to exactly *m*, or to ≤m is somewhat equivalent to the distinction between counting the states inside the volume of phase space and inside a shell of infinitesimal thickness. A result here may shed some light into the related question of negative temperatures [16,17,18,19,20,21].

A word of caution is in order here. In reviewing some of the mathematical proofs, we have noticed how crucial the asymptotic limit in *m* is. For example, in the proof of Erdös and Lehmer [39], we have noticed that there are terms that are neglected as being o(1) in *m*. However, they are as large as $10^{15}$ at their maximum, where *m* is around $10^{12}$. They are still as large as $10^{13}$ when *m* is approximately $10^{15}$. It takes *m* to be circa $10^{18}$ before unity is approached. Those and many other terms are routinely neglected because they eventually converge to zero for large *m*. This ‘finite size’ effect is partly mitigated by the fact that the entropy is logarithmic in the total number of partitions. At the same time, many proofs require that a tremendous number of such terms are neglected. For the physics of quantum systems of small size, there could be new insights emerging which could be traceable to such mathematical subtleties. For classical systems, one is often content with Stirling’s formula, which is a much less troubling approximation.

For massive bosons, we have thus pointed out that there must be a special threshold at which the number of particles (*N*) is of the order of the energy quanta (*m*) to the power of 2/3 (one dimension) or 3/5 (three dimensions). This is the ‘optimal’ number of particles that maximizes the number of partitions to obtain exactly *m* quanta. We have characterized the entropy and the fluctuations near that region. It is only when the number of particles is smaller than such order that the entropy becomes more concisely *N*-dependent, since *N* is now a genuine limiting factor. We have found foreseeable limitations. For example, the entropy as an explicit function of both the energy and of *N* is not available, in a uniform sense, i.e., without focusing on very small (N,m) regions. There could be problems concerning small systems that may not be attackable with the traditional approach, for example starting from the grand canonical ensemble. In fact, both the canonical and grand canonical ensembles may hide subtleties originating in the finiteness of *m* and *N*. This could be a problem even in ‘equilibrium’ systems, of mesoscopic size, or for weakly perturbed systems.
